# “Such conversations are not had in the families”: a qualitative study of the determinants of young adolescents’ access to sexual and reproductive health and rights information in Rwanda

**DOI:** 10.1186/s12889-022-14256-9

**Published:** 2022-10-07

**Authors:** Valens Mbarushimana, Daphney Nozizwe Conco, Susan Goldstein

**Affiliations:** 1 School of Public Health, Faculty of Health Sciences, University of the Witwatersrand, Johannesburg, South Africa; 2 School of Public Health, College of Medicine and Health Sciences, University of Rwanda, Kigali, Rwanda; 3 SAMRC/Centre for Health Economics and Decision Science, PRICELESS SA, School of Public Health, Faculty of Health Sciences, University of the Witwatersrand, Johannesburg, South Africa

**Keywords:** Gender, Rwanda, Sexual and reproductive health and rights, Sexuality education, Young adolescent

## Abstract

**Background:**

Access to sexual and reproductive health and rights (SRHR) information during adolescence has become a global concern. This study explored factors that enable or prevent young adolescents from accessing to SRHR information from the perspective of the key informants in Rwanda.

**Methods:**

We conducted a qualitative study using semi-structured interviews with 16 purposively selected key informants from public and private institutions in Rwanda. This selection was based on their positions and expertise in delivering SRHR information to adolescents. The interview guide questions were designed based on the social-ecological theoretical framework of adolescent health. The interview transcripts were recorded, transcribed, translated and thematically analysed in Nvivo 11.

**Results:**

The study reflected that multiple enablers and barriers at the individual, relationship, community and societal levels determined young adolescents’ access to SRHR information. These determinants include information-seeking behaviour and age of starting sexuality education at the individual level; and parents’ limited communication with young adolescents due to taboos, lack of skills, limited parental availability, beliefs, lack of appropriate language and peer norms at the relationships level. Enablers and barriers at the community level were the diversity of SRHR sources, the scope of sexuality education programmes, and cultural and religious beliefs. Finally, the perceived enablers and barriers at the societal level consisted of inadequate resources, inappropriate SRHR policy-making processes and unfriendly SRHR laws.

**Conclusion:**

Enabling access to SRHR information requires addressing multiple factors within the social-ecological environment of young adolescents. Addressing these factors may facilitate improved access to SRHR information for this age group.

## Background

Access to sexual and reproductive health and rights (SRHR) information during adolescence is a basic human right [1] that helps adolescents to fully develop their potential [2]. The International Conference on Population and Development (ICPD) held in Cairo in 1994 called for the comprehensive sexuality education (CSE) of adolescents to respond to their need for information and skills for a better adolescent SRHR [3]. CSE aims to deliver information on human sexuality and sexual and reproductive health (SRH), gender equity, and human rights, and to help adolescents adopt safe and responsible sexual behaviours [3]. Timely access to high-quality SRHR information plays a central role in adolescents' healthy behaviours and safer sexual practices [4]. Goal 3, targets 3–7, of the Sustainable Development Goals seeks to ensure universal access to SRHR information and the integration of SRH into national programmes by the year 2030 [5]. Research indicates that adolescents wish to receive SRHR information, but that available SRHR information sources may not necessarily be acceptable to adolescents [6]. However, despite the benefits from SRHR information, adolescents experience limited access to SRH information, resulting in poor SRH knowledge, early marriage, pregnancy, and poor decision-making in SRH matters [7].

Young adolescents need accurate information about the biological, socio-cultural, psychological, relational and spiritual dimensions of sexuality [8]. Early adolescence provides a window of opportunity to intensify the delivery of such SRHR information [9, 10]. Through this socialisation process, young adolescents become significantly aware of their bodies, gender and sexuality [11]. Family members, media, and social institutions teach young adolescents appropriate behaviours [12, 13] and norms for interacting, forming relationships, and engaging in social and sexual behaviours [14]. These learned sexual behaviours may have implications on their future sexual health [12].

It is important to understand the layers of young adolescents’ access to SRHR information. Sexuality socialisation processes occur in young adolescents' socioecological environment [10]. This large environment consists of interrelated factors at individual, relationship, community, and society levels [15]. In the current study, we used the socioecological framework of adolescent SRH [16] to understand young adolescents’ access to SRHR information in Rwanda.

Access to information in Rwanda has significantly improved over the last decade. There has been an expansion in media and information and communication technology, and increased licensed radio broadcasts, shows on television stations, internet subscriptions, and mobile telephony penetration [17], indicating an improved access to information for the general population, including young adolescents. Furthermore, Rwanda has created different policies to promote young adolescents’ health [18–20] and put youth friendly corners in health facilities to educate young people about SRH [21]. In addition, CSE has been delivered in primary and secondary schools since 2016 [19, 22] with the aim to increase knowledge of sexuality, gender, and other reproductive issues, including sexually transmitted infections [23].

Despite the significant efforts to educate adolescents on SRHR in Rwanda, young adolescents experience adverse sexual health outcomes. Five percent of female adolescents and 10% of male adolescents engage in sexual activity before they are 15 years old [24]. Pregnancy among schooling girls (10–18 years) remains a countrywide concern [18]. Furthermore, previous studies indicate that young people lack adequate SRHR knowledge; and social prohibition prevents them from obtaining information on SRH [25] and HIV infection [21]; and importantly, adolescents face cultural mores and religious beliefs as major barriers to SRH services [21], including SRHR information.

While the global developmental agenda focuses on enabling universal access to SRH information [3, 5], extant literature does not have sufficient evidence on this aspect among young adolescents. Generally, research involving young adolescents in low- and middle-income countries, including Rwanda, is scant [11, 26]. Studies involving young adolescents have focused on gender norms [9, 27, 28] and attitudes [14]. Assessing sexual wellbeing during early adolescence, Kagesten et al. found that SRHR knowledge and communication were generally low in urban Indonesia [11]. Koenig et al. assessed SRH communication patterns in early adolescence and found that socioecological factors such as older age and pubertal onset are associated with SRH communication [29]. Bankole et al. studied sexual knowledge and information sources for very young adolescents in four sub-Saharan countries. They found that young adolescents lack deep knowledge about pregnancy and HIV prevention and access to multiple SRHR sources of information [30]. Finally, Juariah conducted a baseline survey to assess the reproductive health knowledge of young adolescents and found that their knowledge was poor [31]. It has been acknowledged that access to SRHR information improves sexual knowledge [32, 33] and leads to positive SRHR outcomes [34]. However, there is no clear evidence in the literature on the determinants of access to SRHR information among young adolescents. This study seeks to contribute to existing evidence on SRHR for young adolescents, specifically on the determinants of access to SRHR information for this age group. From a social-ecological perspective, this study aimed to explore key informants’ views on the determinants of accessing SRHR information among young adolescents in Rwanda.

## Methods

### Study setting

We conducted this study in Rwanda, a landlocked country located in Eastern Africa, with a surface area of 26,338 square kilometres. Rwanda is bordered by Uganda in the north, Tanzania in the east, Burundi in the south, and the Democratic Republic of Congo in the west [35]. The Fifth Rwanda Population and Housing Census of 2016 indicated that 52% of the population is younger than 20 years, with young adolescents (10–14 years) representing 13.7% of the general population [36]. The Rwandan population is mainly rural (83%) [37]. The age of sexual consent and legal marriage for all people is 18 and 21 years, respectively [38].

### Study design

This study employed qualitative research methods. Semi-structured interviews with key informants was used to explore their views on the enablers and barriers to young adolescents’ access to SRHR information.

### Sampling and recruitment of participants

The study used a purposive sampling strategy to recruit participants who work for institutions that disseminate SRHR information to young adolescents and youths in Rwanda. The researchers contacted 19 relevant institutions through official correspondence. In total, 16 participants from these institutions were available for interviews after several attempts by the researchers to reach their institutions. These participants included nine (six females and three males) from public institutions, six participants (five males and one female) from faith-based organisations (FBOs), and one female participant from a non-governmental organisation (NGO). The public institutions involved in this research were the Ministry of Education; Ministry of Gender and Family Promotion; Ministry of Sports and Culture; Ministry of Health; Rwanda Biomedical Centre; Rwanda National Children’s Commission; Rwanda Education Board; and the urban districts of Gasabo and Nyarugenge. The FBOs that provided participants included the Roman Catholic Church, the Seventh-Day Adventist Church, the Pentecostal Church, and the Rwanda Muslim Community.

Based on their availability for interviews (as provided by their institutions), we recruited participants who consented to the study. We did not include participants from institutions that did not respond to the researchers' request for data collection after repeated follow-ups.

### Data collection

Data collection was conducted between July and October 2019. Upon approval by the participants' institutions, the researchers made the necessary arrangements to contact participants and determine the modalities of the interviews. Before the interviews, we provided participants with the study information and time to ask relevant questions. Next, researchers invited each participant to sign an informed written consent form. Finally, with the participants’ consent, the researchers digitally audio recorded the interviews. The interview language was Kinyarwanda for 15 interviews and English for one interview. Each interview lasted between 30 and 60 min. The interview guide used during the interviews covered several topics, including participants' views on enablers and barriers to accessing SRHR information among the target age-group population.

### Data management and analysis

**Fig. 1:**
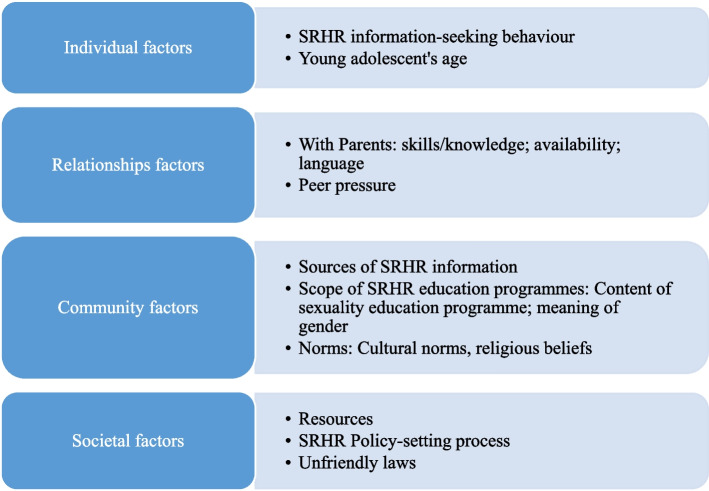
Coding scheme for perceived enablers and barriers to young adolescents' access to SRHR information

The audio-recorded interviews were transcribed verbatim in Kinyarwanda, and professional translators translated these transcripts into English. We used QSR Nvivo 11 (QSR International Pty) for Windows to analyse the data. Using the social-ecological framework of adolescent health, we conducted deductive analysis of perceived enablers and barriers to young adolescents’ access to SRHR information. Two researchers repeatedly read all the interview transcripts independently to familiarise themselves with the contents, and they coded the same data and compared their coded outputs to identify similarities and differences. Common agreement between these researchers allowed resolution of discrepancies in data coding. Next, the two researchers started creating and condensing meaning units, which were later organised into codes. These codes were then organised into sub-themes, which were finally organised into the levels of the social-ecological framework of adolescent health, namely individual, relationship, community and societal levels (Fig. 1) [16]. Finally, we presented the findings in a narrative form and used quotations to illustrate the data description at each framework level.

## Results

### Perceived determinants of young adolescents’ access to SRHR information

We asked the participant to reflect on the determinants of access to SRHR information for young adolescents. The analysis of their responses yielded several factors at individual, relationship, community and societal levels of adolescent health.

### Individual factors

The participants reflected on two determinants of accessing SRHR information for young adolescents at the individual level. These determinants were adolescents’ information-seeking behaviour and the appropriate age for starting sexuality education.

### SRHR information-seeking behaviour

The participants discussed several factors related to young adolescents’ access to SRHR information at the individual level. The participants reported that young adolescents are curious and seek sexual health information from various sources. Nevertheless, the study participants noted that young adolescents are often unable to satisfy their curiosity by asking their parents and thus harbour misconceptions. The participants thought that young adolescents feel shy to ask questions and think their parents consider questions disrespectful. In addition, participants felt that young adolescents do not know where to obtain the right SRHR information despite their curiosity about SRHR. One participant said the following about what influences young adolescents access to SRHR information at the individual level:

*“They [young adolescents] do not know they need information. When we talk to them at school, we find out that they are curious, but they give us some wrong information about sexual and reproductive health. They do not know that they need the right information”. (*P16, male, public institution)

In addition, participants said that young adolescents are scared to ask for SRHR information from their parents and look to the celebrities they choose as their role models and around whom they build their identity. Some participants illustrated why young adolescents seeking information from their parents as follows:

*“They [young adolescents] are not comfortable discussing their reproductive issues. They believe their parents will consider them as disrespectful, bad-mannered people, and uneducated”. (*P10, female, public institution)

*“They [young adolescents] search for information about football or movie stars to know them better and choose them as their role models”. (*P01, female, public institution)

### Age of initiating SRHR education

The participants indicated that it is important to start receiving SRHR education during early adolescence, but they did not specify the exact age. When asked about the age at which sexuality education should be initiated, some participants felt that it should begin as early as possible, and others suggested that SRHR education should be initiated at ten years of age. Responding to the question about the exact age to initiate SRHR education, some of the participants said the following:

*“Even before they grow into young adolescents, they should be taught about sexuality according to their developmental age.”* (P01, female, public institution)

*“We realised that at ten years, children are curious and start being exposed to those challenges of sexual and reproductive health information. So parents can prepare their children by starting to talk to them about sexuality."* (P16, male, public institution)

### Relationship factors

The participants noted that access to SRHR by young adolescents is influenced mainly by two factors at the relationships level namely communication between parents and young adolescents, and peer influences.

### Parent–child interaction

The findings indicated that sexuality is not a topic that is discussed between children and their parents for several reasons. They reflected that it is culturally inappropriate for parents to deliver sexuality information to children because sexuality is a taboo topic and has been for many generations. In addition, parental reluctance to engage with children about SRHR information is due to the belief that they may start behaving badly once they are informed. According to the participants, this attitude comes from the cultural belief that SRHR education encourages children to initiate sexual activity. Two participants explained why parents do not communicate adequately with their children on SRHR as follows:

“*The Rwandan culture does not encourage parents to discuss reproductive health with children. Many people don’t talk about it. It is a taboo subject. It is due to the way people are raised.” (*P10, female, public institution)

“*People think that giving information to children means encouraging them to have sex.*” (P16, male, public institution)

One participant insisted that parents send young adolescents to other family members (such as aunties) for specific sexual information, which proves that sexuality cannot be discussed at the family level. We asked participants with whom such discussion takes place, and one participant answered as follows:

*“Sending young adolescents to their aunts proves that sexuality cannot be discussed at the family level.”* (P14, female, public institution)

Furthermore, the participants recognised that parents' knowledge and attitudes play a key role in delivering sexuality education to young adolescents. They noted that some parents do not provide sexual information to young adolescents because of their own limited knowledge, which is explained by one participant in the quotation below. However, another participant reflected that some educated parents are able to communicate with their children on SRHR:

“*Parents do not have enough information. Sometimes we ask them to teach their children but they don’t have that knowledge either.” (*P14, female, public institution)

“*Parents' education affects their children's sexuality knowledge. Educated parents with open discussions have well-informed children.”* (P12, male, FBO)

Parents’ availability is another important factor to access to SRHR information for young adolescents. the participants perceived that changes in family living conditions have resulted in parents being absent from their home and in the use of housekeepers who cannot adequately address children's SRHR information needs. All participants noted that parents do not have enough time to engage with their children because of limited parental availability. According to the participants, parents might discuss sexuality if they spend more time with their children. One of the participants shared the following:

“*I have noticed that various topics can come up when parents talk to their children for a long time. However, when time is short, they do not have the means to discuss sensitive topics such as sexuality. Parents come home tired and asking if their children did their homework and had proper meals.*” *(P08,* male, FBO)

Language, as a medium of discussion, was another concern for providing SRHR information to young adolescents, specifically the lack of appropriate words to speak about genital parts in Kinyarwanda. The participants noted that words for genitals are perceived as very rude, uncommon, and hard to say to children. Therefore, they indicated that they use alternative words in Kinyarwanda, which can lead to confusion. Nevertheless, the participants said they were comfortable talking about sexuality in foreign languages such as French or English. The participants further mentioned a booklet to guide SRH communication with young adolescents titled *‘Tuganire Mwana Wanjye’* (Let’s Talk, My Child) that can help in parent–child discussions on sexuality, and thus, help parents find the appropriate words in Kinyarwanda. The participants said the following about the language and available ways to overcome the language challenges:

“*For instance, saying the word 'penis' in Kinyarwanda is considered a problem, but it sounds fine in French or English.*” (P16, male, public institution)

“*We developed that book to encourage parents to overcome fears about discussing [sexuality] with their children. The book aims to give parents words they can use while talking to their children and help them to face some of the problems ahead as they grow up.”* (P01, female, public institution)

### Peer pressure

The participants perceived peer pressure as another determinant of young adolescents accessing SRHR information. According to this study, peers play a significant role in delivering SRHR information to young adolescents, especially about sexual relationships. The participants noted that male and female adolescents have different expectations, and boys perceive girls as money spenders while girls consider boys as providers and hunters for sex. According to the participants, these norms mean that girls need to have sex if they want to get any money or presents from boys. A participant explained it as follows:

*“Boys should always pay everything for the girl. ... if he spends money on a girl according to the norm, she should also pay him back. Therefore, he expects that pay to be sex." (*P02, female, NGO)

In addition, the participants thought that peer pressure contributed to spreading SRHR information that contradicts the social expectations of girls about remaining virgins until marriage by expecting them to keep their boyfriends by proving love through sexual intercourse. They also revealed boys are entitled to have multiple sex partners. A participant illustrated this contradiction between peer and social norms as follows:

“*Peers spread messages that are in contrast with the social culture or expectation of being a virgin until marriage. For example, ‘a real boy has sex with multiple girls’; or similarly for a girl, ‘you’re not a real girl until you lose your virginity’.”* (P13, female, public institution)

### Community factors

The determinants of access to SRHR information among young adolescents included the sources of information, the scope of SRHR education and various cultural and religious norms.

### Sources of SRHR information

This section presents participants’ perspectives on various sources as enablers of access to SRHR information for young adolescents. The participants indicated that young adolescents access SRHR information through multiple sources and that these sources have diversified because of technological advancement. Some of the sources that emerged include mobile phones and television. When asked about which sources are delivering SRHR information, one participant FBO outlined them as follows:

“*They [young adolescents] get such information from their friends, parents. Schools deliver such information as well. They also access information through information and communication technology, the internet, mobile phones, YouTube, and others.”* (P03, female, FBO)

Furthermore, the participants reported that young adolescents seek SRHR information from social media sources, including YouTube, Facebook, WhatsApp, and the internet. However, they noted that some internet sources spread incorrect SRHR information, and young adolescents cannot differentiate between correct and incorrect information. The participants further highlighted the disparities in SRHR sources between rural and urban settings and stated that young adolescents from the urban areas have social media as their main sources of information. In contrast, young adolescents from rural areas rely on schools to obtain SRHR information. Some of the participants said the following:

“*In towns, they get information from social media…, in the village, I would not put social media on the first place. I would say that they get information from schools*.” (P12, male, FBO)

“*The reality is that they access right and wrong information and they are not good at deciding which information is okay and which is not*.” (P02, female, NGO)

However, given the diverse sources of SRHR information for young adolescents, especially online sources, a participant noted that the Government of Rwanda has established a policy to prevent the misuse of these sources and access to unsuitable information by children. This participant noted the following:

*“There is a policy called Child Online Protection Policy to protect children from abusing the internet.”* (P01, female, public institution)

Some participants noted that various governmental, local and non-governmental leaders offer young adolescents SRHR information, and that it is necessary to consolidate this information from various sources because of the developmental stage of the audience. One of them explained it as follows:

"*Government institutions and non-governmental organisations also prepare different education sessions targeting young adolescents. Organisers need, however, to consider their audience, lifestyles, and the clarity of their messages.”* (P05, male, public institution)

Furthermore, the participants noted that schools are sources of SRHR information through the comprehensive sexuality education (CSE) curriculum. In addition to the CSE curriculum, the participants noted that young adolescents learn about SRHR through gender and health clubs. One participant explained the role of school clubs in delivering SRHR information as follows:

“*There are after-class clubs that teach about gender principles and reproductive health. There are also special clubs that focus on health.” (*P17, female, public institution)

In addition to schools, the participants highlighted that trained staff from health facilities deliver SRHR information to young adolescents through youth corners. The role of health facilities was describe by a participant as follows:

“*We trained at least two people in every health centre. The program is in place. Young adolescents receive information on sexual and reproductive health from the youth corner, at the health facility level.”* (P16, male, public institution)

The participants said that radio, television, and mobile phones were other important means of delivering SRHR information to young adolescents. The radio programs include "Urunana” and “Ni Nyampinga”. *For example, Urunana* targets the general population, and *Ni Nyampinga* provides SRHR information that mainly targets young girls. This program calls young girls to abstain from sex or to use condoms. "Mobile for Reproductive Health" (m4RH) is another program through which young adolescents access SRHR information. Using mobile phones, m4RH sends SRHR messages to children. For example, a participant said the following about some of these programmes:

*“They [young adolescents] obtain information from radio programs such as Urunana.”* (P07, male, FBO)

In addition, the participants indicated that churches deliver SRHR information to young adolescents focusing on the ‘Word of God’ and Christian moral values including the avoidance of premarital sex. A participant from a FBO explained it as follows:

“*Churches organise seminars for young children and young adolescents but not regularly. The church's education focuses more on the Word of God than any other topic and calls them to abstain from premarital sexual activity.”* (P03, female, FBO)

### Scope of SRHR education programmes

The study found that the SRHR content is crucial to enable access to SRHR information for young adolescents. Some of the participants were concerned with the content of SRHR education programmes and the meaning of specific concepts.

### Disagreement about the content of SRHR education programmes

The content of SRHR information was seen as crucial to enable access by young adolescents. There was a disagreement between the participants about what to include in SRHR education programmes. We asked the participants what they thought the scope of the education should be. Participants from FBOs focused on moral education and prohibiting premarital sexual activity, and those from public institutions suggested that churches should go further and teach young adolescents about having safe sex using modern contraceptive methods. The following comments show the disagreements between some of the participants:

“*People think different churches should teach young adolescents about moral values, including avoiding illegal sex and promoting abstinence. Telling them to use injections or pills is pushing them into illicit sex.”* (P12, male, FBO)

“*In addition to the natural family planning methods, young adolescents need to be aware of other existing medical contraceptive methods.”* (P10, female, public institution)

“*There are people who only teach that having sex is a sin without explaining more. They do not go beyond that to suggest means for safe sex. We need to avail emergency contraceptives to young adolescents who fail to abstain from sexual intercourse to prevent unplanned pregnancy.”* (P16, male public institution)

The participants noted that the selection of CSE content must respect the culture and get inputs from various education stakeholders. They indicated, for example, that some content in the CSE curriculum, such as the dating scenarios, might affect young adolescents' behaviours, and they believed that dating activities might result in sexual activity if children were not monitored at school and in family environments. In addition, the participants were concerned that involving students in dating scenarios would lead them to discovering things that go against morals and culture. One participant described this concern as follows:

*“Some lessons may invite students into early sexual activity and/or early pregnancy when it comes to in-class dating exercises and reaching an agreement on having safe sex using a condom. It is difficult to protect them from sexual activity in their families or the school environment.”* (P08, male, FBO)

### Meaning of the concept of gender

The participants believed that some concepts such as gender must be taught, but it is difficult to make it meaningful and contextualise it, which makes it difficult to teach to young adolescents. The participants noted that the communities do not clearly understand the meaning and dimensions of gender and believe it has several ideological components that cannot be expressed in Kinyarwanda. Participants highlighted it is difficult to teach ideological concepts to young adolescents who usually learn better from concrete examples, and this makes it hard for teachers to select which content to teach. One participant explained as follows how the meaning of gender influences access to SRHR information for young adolescents:

“*There is still a problem of lack of standard definition of gender. What is the appropriate subject to teach about it? It is still a problem as long as there is no clear definition of gender in our context.”* (P08, male, FBO)

### Norms

The participants reflected on the role of cultural norms in enabling or hindering access to SRHR information by young adolescents. Their views on this aspect are summarized in this section.

### Cultural norms

The participants indicated that social expectations play a significant role in delivering SRHR information to young adolescents. For example, they pointed out that the general expectation is that girls should not talk about having sex and keep silent about their sexual needs, which they perceived as compromising girls’ rights, especially because it prevents them from receiving information on safe sexual relationships from peers. The study also highlighted the existence of other harmful sexual norms. The following comments explain how social expectations influence young adolescents’ access to SRHR information.

“*They [boys and girls] never talk about whether the boy wants to have sex. In some cases, the girl also wants it, and it is her right to say she wants it. The problem is that the general expectation is that a girl should never talk about having sex.” (*P02, female, NGO)

*"Girls grow up shy because culture expects them not to speak in public. Therefore, it is easy for anyone to deviate or fool them because they cannot speak up as the society expects them always to be quiet." (*P14, female, public institution)

The participants noted that cultural norms enhance imbalances in sexual decision-making powers between boys and girls. They reflected that the environment of young adolescents generally promotes boys as protectors of girls and the owners of sexual power, allowing them to initiate sexual relationships. On the other hand, girls have to simulate weakness, play a passive role, and enjoy boys’ protection. It is taboo for girls to express their sexual needs. The following comments discuss this problem:

“*Boys are protectors of girls. A girl who does not surrender to this protection behaves like men and will not find someone to marry her.”* (P01, female, public institution)

“*Boys grow up in an environment that makes them feel that they are the ones to initiate relationships with girls.”* (P05, male, public institution)

*“It is taboo [for a woman] to tell her husband that she wants sexual intercourse, while as a human being, she may also wish to have sex.” (*P01, female, public institution)

In addition, the participants pointed out that the culture plays a role in promoting inadequate SRHR information and creates negative beliefs in young male adolescents. One participant gave an example of a saying that supports the imbalances in sexual decision-making powers and confirms that boys or men cannot control their sexual drive, and therefore, need multiple sexual partners. This may influence young boys’ and girls’ behaviours if they grow up in an environment where such beliefs are held. One participant explained it as follows:

“*A Rwandan proverb says that bulls are irresistible whenever they want sex (*Imfizi ntiyimirwa*). Boys grow up as such and behave like their fathers with such beliefs.” (*P01, female, public institution)

### Religious beliefs

The findings indicated that religious institutions play a role in delivering SRHR information to young adolescents. The study found that FBOs provide SRHR information that influences the sexual knowledge, beliefs, and behaviours of young adolescents. However, some churches are reluctant to teach sexuality because they regard it as a sin. FBOs believe that providing SRHR education promotes immorality and violates God's commandment to abstain from premarital sex. Furthermore, the FBO participants indicated that delivering information on condom use might encourage premarital sex and breach some commandments. Some participants elaborated as follows:

*“The church education focuses on moral values. We teach by good examples of their grandparents, parents, elder brothers, and sisters, the Virgin Mary and other Saints who resisted rape and refused to break their virginity. We teach young adolescents to abstain from having sex, and remain virgins and chaste.”* (P07, male, FBO)

*“Teaching children to use a condom means inviting them to have protected sex. If the commandment says to abstain from sex, it would be a contradiction because that commandment would be violated whether sex is protected or not. It is immoral, misbehaving.”* (P11, male, FBO)

### Societal factors

Three factors at the societal level emerged from the participants’ responses as influencing young adolescents’ access to SRHR information. These factors are related to resources (financial and human), the process of creating SRHR education policies, and SRHR laws.

### Inadequate resources

The study found that inadequate financial resources to generate SRH information tools such as books and training modules are an important challenge to giving young adolescents’ access to SRHR information. The participants also noted that some teaching materials provided by partners such as non-government organizations (NGOs) do not adequately match young adolescents’ needs. They further pointed out that the shortage of skilled and qualified personnel and the inability to use available communication channels, including social media affected the provision of SRHR information to young adolescents. Some participants said the following about the challenges to enabling access for young adolescents to SRHR information:

“*It [sexuality education] also requires financial means. Books are expensive... Modules require time to generate.”* (P17, female, public institution)

“*Schools have no adequate materials apart from those found in biology, which are not also from our culture. They are most often brought from the NGOs. The package from NGOs is always wrong.” (*P08, male, FBO)

“*We have a shortage of employees to deliver sexuality information….Another barrier is technology use. We want to send that information using WhatsApp and other social media, but it is still expensive and children do not access it on phones*.” (P16, male, public institution)

### Inadequate SRHR policy-setting process

The participants criticised the processes of creating SRHR policies as inadequate, as they do not involve people from all levels of communities, including the older adults. The findings indicated that it is necessary to create evidence-based policies through research. They noted that some of these policies originated from external sources and have no local relevance. Two participants explained it as follows:

“*The family members, especially elderly people from the villages, are not consulted. If they [policy-makers] could approach them, they would learn about what used to happen 20 years ago, how women behaved towards their husbands and how the husbands behaved towards their wives, and what made them keep their family secrets.” (*P05, male, public institution)

“*Policy content should not be imposed. Policy-makers abruptly come up with content from European countries. They do not research to get informed about the local context to include community members’ cultural perspectives.”* (P08, male, FBO)

### Unfriendly SRHR laws

The participants indicated that some laws do not facilitate access to SRHR information for young adolescents. One participant noted that it is necessary to solve specific reproductive health problems and revise SRHR laws that do not allow young adolescents to access information on SRHR services, such as emergency contraceptives. One participant explained it as follows:

“*We should also revise current laws to support children who have difficulty to abstain from sex and need emergency contraceptives.*” *(*P16, male, public institution)

## Discussion

Informed by the social-ecological framework of adolescent health, this research sought to explore participants’ perspectives on the enablers and barriers to young adolescents’ access to SRHR information in Rwanda. The social-ecological framework describes multiple interacting levels of interventions to enable adolescent SRHR [16], especially access to SRHR information by young adolescents. Participants in this study perceived that access to SRHR information for young adolescents consists of interrelated individual, relationship, community and societal factors.

The study found that individual factors include the young adolescents' SRHR curiosity and age determining the exact time to initiate sexuality information. The study found that young adolescents ask SRHR related questions to know more about SRHR but these questions show they have inadequate SRHR knowledge and suggest their need for more knowledge. Previous research also highlighted that young adolescents in Rwanda receive partial and inaccurate SRHR information [39]. A recent scoping review concluded that young adolescents in low- and middle-income countries have inadequate SRHR knowledge, especially in terms of puberty and menstruation [40]. Young adolescents’ SRHR knowledge in Rwanda is inadequate, as in many other settings such as Sub-Saharan Africa [30, 41], Guangzhou City in China [42], Korea [43] and Indonesia [11]. The age of initiating sexuality education was another perceived determinant of access to SRHR information by young adolescents. However, participants did not agree on the exact age at which to deliver such education. Kingori et al. suggest that the age of receiving SRHR information may influence people’s sexual health education [44] and other literature suggests that sexuality education must be age-appropriate and start at an early stage [3], specifically in early childhood [45]. Sometimes, young age is considered a barrier to sexuality communication [46] because it is thought that it is too early for young adolescents to receive SRHR information [46, 47], but other studies conclude that young adolescents have insufficient SRHR knowledge [43].

The relationship factors perceived to influence access to SRHR information included parent–child interaction and peer norms. Similar to other studies [48], the current study found that sexuality is not openly discussed with parents but that it is sometimes discussed with members of the extended family [49]. However, young adolescents often perceive parents as the primary sources of SRHR information [49, 50], but many parents do not discuss sex with their children. There is limited communication with parents because parents lack the skills and time to engage with young adolescents, have negative attitudes towards SRHR information, and face language-related barriers and cultural norms at the family level. In other studies, parents also had limited resources, including knowledge [48], negative attitudes [48] and socio-cultural norms [48, 51] as barriers to SRHR communication with (young) adolescents. The belief that parents are worried that SRH education will stimulate sexual activity among young adolescents is echoed in other studies [46, 52]. Usually, parents perceive sexual activity as bad, shameful, and morally reprehensible, which impedes the discussion of sexual health topics with young adolescents [48]. The study found, like other studies, that parents send young adolescents to members of extended family (aunts, uncles, and grandparents) for sexual socialisation [12, 47, 53].

The participants thought that educated parents are more likely to discuss sexuality with young adolescents than uneducated parents. The influence of parental education was reported by another study in Iran whereby the mother's education influenced young adolescents' SRHR knowledge [54]. In addition, while young adolescents need adults who spend time with them [47, 55], this study shows that parental absence from the home reduces parental monitoring and opportunities to deliver SRHR information. Furthermore, this study revealed that the language to engage with young adolescents about sexual information in local terms is culturally inappropriate and embarrassing. However, a booklet in the local language, Kinyarwanda, is available to facilitate the SRHR communication between parents and young adolescents [56].

Similar to a previous study in Tanzania [57], this study highlighted the role of peers in delivering SRHR information. Peer norms do not allow girls to fully access SRHR information and encourage silence about sexuality, and this is similar to findings from Vietnam [58] and Ethiopia [47]. Silence about SRHR implies that young adolescents do not have access to a full range of SRHR information, which might affect girls' SRHR information-seeking behaviour [47]. Furthermore, the SRHR messages from peers are contradictory, calling for both sexual abstinence and intercourse. This finding aligns with previous research indicating (young) adolescents follow contradictory peer norms [59].

Access to SRHR information requires using relevant sources at the community level. Echoing other studies [30, 48], this study revealed that young adolescents access SRHR information from numerous sources. The current study found that structured programs such as school-based sexuality education, youth-friendly corners at health facilities, *Urunana* and *Ni Nyampinga radio shows,* and (m4RH) provide reliable SRHR information to young adolescents. However, the study found that schoolteachers face limitations such as beliefs, attitudes, and lack of skills when delivering SRHR information. In addition, some sources provide SRHR information based on the gender of the recipients. For example, *Ni Nyampinga* is created by girls for girls, and this radio show targets only girls, provides information about health and relationships and depicts ideal girlhood for young female adolescents [60, 61]. m4RH is a text message-based platform that involves sending SRH information to young adolescents in Kinyarwanda [62]. However, m4RH is reaching a limited number of young adolescents because of low mobile phones ownership by young adolescents. Furthermore, the findings suggest that young adolescents access less reliable and unprofessional SRHR information sources, including friends, social media platforms (Facebook and WhatsApp) and mobile phones, which raises concerns about the overall quality of messages delivered and has led to calls for SRHR information harmonisation.

The study highlighted the influence of culture and gender norms on delivering SRHR information to young adolescents because of taboos. The study corroborated findings from other studies that sexuality is a taboo and embarrassing subject to discuss with young adolescents in many societies [52]. Sexuality taboos also influence the design and implementation of sexuality education programmes [63]. Sexuality taboos suggest that providing and seeking sexual information sounds unacceptable to many young adolescents.

Like other studies [12, 64], the current study found differences in boys’ and girls’ gender roles, suggesting a different socialisation process that affects access to SRHR information. We found that the community expects girls to remain submissive, ambivalent or silent about sexual matters, but that boys are expected to enjoy their sexual powers and freedom; this is also reported in previous studies [47, 65].

In addition, the findings from this study suggest that religious beliefs play an important role in determining the content of SRHR information in Rwanda [21], which is similar to other studies [66–68]. Participants from FBOs espoused for preserving morality among young adolescents by focusing mainly on the moral values of sexual abstinence and virginity. In contrast, participants from public institutions advocate for safe sex and modern contraception. These findings suggest that, like another study [69], all education stakeholders must suggest consistent content of SRHR information. In addition, the study found there is no agreed-upon definition of the concept of “gender” applicable to the Rwandan context. Gender has a significant influence on sexuality [70]; hence, well-defined content serves as a foundation for determining what, how, when, and to whom to best to provide it [71].

This study found that factors at the societal level also influence access to SRHR information. These factors include inadequate resources, policy-setting processes and SRHR laws. Concerning the resources, the study noted, like others [72], limited means to develop teaching aids and a shortage of trained employees (including schoolteachers) to deliver SRHR. In this regard, inadequate resources constitute challenges to implementing sexuality education in Rwanda [21] and other resource-poor settings [73–75].

This study also identified the challenges of inadequate SRHR education policy-making processes and laws that do not involve local stakeholders. Participants highlighted the need for research before initiating SRHR policy-making and involving local stakeholders. These findings underscore the perceived importance of research and involvement of stakeholders from different backgrounds to come up with a universal and culturally-relevant SRHR content [69]. Additionally, the findings from this study suggest the need for revising adolescent SRHR laws to enable access to SRHR services and information by young adolescents without parental consent as recommended by the World Health Organization [76].

It is crucial to consider the following limitations while interpreting the findings from this study. First, the results could have been enriched by perspectives from various participants, including young adolescents' parents, healthcare providers and schoolteachers; hence the limited generalisation of the findings. Furthermore, the results rely solely on the stakeholders' perspectives using a qualitative approach. Quantitative studies are important to ascertain the importance of each of the discussed factors in supporting or hindering access to SRHR information among young adolescents. Despite these limitations, the study gives insights from a wider perspective on the factors that influence access to SRHR information among young adolescents in Rwanda.

## Conclusion

This study showed that many of the participants had a favourable attitude towards young adolescents’ access to SRHR information. Informed by the social-ecological framework of adolescent health, this study provided an overview of enablers and barriers to access to SRHR information by young adolescents at individual, relationship, community and societal levels. Addressing these barriers may facilitate improved access to SRHR information for young adolescents. These findings are important to promote safe SRH and preventing adverse health outcomes among younger and older adolescents.

## Data Availability

The datasets generated and/or analysed during the current study are not publicly available to preserve the anonymity of the research participants but are available from the corresponding author on reasonable request.

## Abbreviations

ADEPR: Association of Pentecostal Churches of Rwanda
CSE: Comprehensive Sexuality Education
FBO: Faith-Based Organisations
ICPD: International Conference on Population and Development
m4RH: Mobile for Reproductive Health
NGOs: Non-governmental organisations
SRH: Sexual and reproductive health
RBC: Rwanda Biomedical Centre
SRHR: Sexual and Reproductive Health and Rights
